# Development and validation of “parental satisfaction with knee-to-knee dental examination” scale: high correlation with parental sense of competence

**DOI:** 10.3389/froh.2026.1822153

**Published:** 2026-05-01

**Authors:** Avia Fux-Noy, Keren Ytshaki, Yael Raviv, Gal Harpaz

**Affiliations:** 1 Faculty of Dental Medicine, Hebrew University of Jerusalem, Jerusalem, Israel; 2 Department of Pediatric Dentistry, Hadassah Medical Center, Jerusalem, Israel; 3 Department of Education and Psychology, The Open University of Israel, Raanana, Israel

**Keywords:** knee-to-knee position, parental satisfaction, parental sense of competence, scale development, validation

## Abstract

**Introduction:**

The knee-to-knee position is considered an effective and comfortable position for patients, parents, and dentists to facilitate a toddler's dental examination. Since the knee-to-knee method requires the involvement of parents, greater parental competence may facilitate more comfortable involvement in the examination and may influence parents' satisfaction. The objectives of the study were (1) to develop and validate a scale measuring parental satisfaction with the knee-to-knee dental examination position (2); to assess parental satisfaction with the knee-to-knee dental examination; and (3) to examine the relationships between parental competence, the dentist's estimation of parental satisfaction, and other demographic characteristics and parental satisfaction.

**Methods:**

A 13-item questionnaire measuring parental satisfaction with knee-to-knee dental examination (PSKTK) was developed. The questionnaire was validated via two samples to perform exploratory factor analysis and confirmatory factor analysis. Parental satisfaction was subsequently contrasted with other key variables of interest, such as parental sense of competence (PSOC), dentist's estimation, age and gender. Differences in the main variables were tested.

**Results:**

An 11-item version of the PSKTK scale was validated. Parental satisfaction was found to be high in 90% of sample 1 and 85% of sample 2. Parental satisfaction was significantly correlated with PSOC (*p* =.003 for sample 1, *p* < 0.01 for sample 2) and with dentists' estimation of parental satisfaction (*p* < 0.001 for sample 1, *p* < 0.01 for sample 2).

**Discussion:**

The 11-item questionnaire was found to be valid and reliable and can be used to assess satisfaction with knee-to-knee positions among different populations. Parents of toddlers are satisfied with this examination position, especially those with a greater sense of parental competence.

## 1 Introduction

Establishing a dental home to infants and their families provides an opportunity to educate parents about their children's oral health. The American Academy of Pediatric Dentistry (1), the American Dental Association (2), the European Academy of Paediatric Dentistry (3) and the International Association of Paediatric Dentistry (4) recommend that a child's first dental visit and oral examination occur by 1 year of age (1–4).

Various positions are possible for facilitating a toddler's dental examination. The knee-to-knee position is considered an effective and comfortable position for the patient, parent, and dentist (5–7). In this position, the dentist and parent seat face to face, and the infant is placed on the parent's lap, facing the parent, with the legs wrapped around the parent's waist. While the parent holds the child's hands, the child is laid back, resting the head in the dentist's lap. This position enables the child to see and feel the parent while the dentist performs the examination. The direct involvement of the parent provides emotional support to the child and enables the parent to help restrain the child (5–7).

Surveys among pediatric dentists reported that 91%–97% of respondents used knee-to-knee position for dental examinations (8, 9). In addition, 88% of Israeli respondents estimated that parents were satisfied with this examination method (9). However, information regarding parents' satisfaction with the knee-to-knee approach is lacking. Measuring parental satisfaction across various domains in pediatric dentistry is common, using questionnaires and Likert scales (10–13). Evaluating this satisfaction can assists dentists in choosing the preferred clinical or behavioral approach.

Parental sense of competence (PSOC) is defined as the parent's assessment of their ability to function across a variety of tasks related to the demands of the parental role. These perceptions and beliefs serve as a primary cognitive “key” to adaptation and parental ability to interact effectively with their child (14). Studies have shown that there is a correlation between parental competence and parental and child behavior (15). Since a dental examination using the knee-to-knee method requires the involvement of parents, greater parental competence may facilitate more comfortable involvement in the examination and may influence parents' satisfaction.

Thus, the objectives of this study were (1) to develop and validate a scale to measure parental satisfaction with the knee-to-knee dental examination position (2); to assess parental satisfaction with the knee-to-knee position; and (3) to examine the relationships between dentists' estimation of parental satisfaction, parental competence, and other demographic characteristics and parents' satisfaction. The study hypotheses were that parents would report high satisfaction levels, which would correlate with the satisfaction estimated by the examining dentists, and that higher PSOC scores would correlate with greater parental satisfaction.

## 2 Materials and methods

### 2.1 Scale development- parental satisfaction with knee-to-knee dental examination (PSKTK) scale

The questionnaire was developed in Hebrew for the purpose of this study (attached as supplementary file) by two researchers: a specialist in pediatric dentistry and a social psychologist. Thirteen initial statements were developed by a panel of five pediatric dental experts, with prior research experience in this area to establish high content validity, covering four domains: direct visualization of the oral cavity for both the dentist and the parent, understanding, safety and comfort, and professionalism. These items covered all key domains associated with the knee-to-knee position, as identified in the literature (5–7) and through expert clinical experience. The accompanying parent indicated level of agreement with each statement regarding the examination position using a 5-point Likert scale, ranging from 1 (do not agree at all) to 5 (strongly agree). Each participant received a mean score for the 13 items. A score of 1–2.99 reflects low satisfaction, 3–3.99 reflects moderate satisfaction, and 4–5 reflects high satisfaction. In addition, a 5-item questionnaire was developed by the experts panel to be completed by the examining dentist. This instrument, titled the “Dentist's Estimation of Knee-to-Knee” (DKTK), measured parents' satisfaction as perceived by the clinician. The items were phrased similarly to those in the PSKTK, and a mean score was calculated for each participant. This scale was utilized as a supplementary variable and did not undergo a formal validation process. All the participating dentists were pediatric dentistry residents skilled in the knee-to-knee examination method. All the participants underwent uniform training to standardize the completion of the DKTK questionnaire.

### 2.2 Parental sense of competence (PSOC) scale

The PSOC scale was first developed by Gibaud-Wallston (16) for parents of preschool children. Later, Johnston and Mash (17) expanded it for parents of older children. The questionnaire comprises two subscales, one with 9 items measuring satisfaction with parenting and the other with 8 items measuring parenting competence. In the present study, the Hebrew version (18) was used for the subscale of parental competence only. The accompanying parent rated the extent of their agreement or disagreement with each statement on a scale from 1 (do not agree at all) to 6 (strongly agree). Each participant received a mean score for the 8 items. A score of 1–2.99 reflects low competence, 3–4.99 reflects moderate competence, and 5–6 reflects high competence.

### 2.3 Study group

The study was conducted in the Department of Pediatric Dentistry. A convenience sampling was employed, recruiting parents of children up to 4 years of age attending the dental clinic during the study period, and the children were examined in the knee-to-knee position. Participation was entirely voluntary. The inclusion criterion required proficiency in the Hebrew language. The exclusion criteria were as follows: the child arrived accompanied by a caregiver other than one of his parents, and the accompanying parent did not understand or read Hebrew. For each participant, the following data were collected about their visit to the clinic: child's age and gender, parent's age and gender, and the dentist's gender. Additionally, parents provided an estimation of the child's dental anxiety by rating the extent to which the child approached the clinic “happily.” Finally, it was documented whether this was the child's first dental visit. The parents completed the PSKTK and PSOC questionnaires, and the examining dentist completed the DKTK questionnaire and scored the child's behavior during the examination according to the Frankl behavior rating scale.

### 2.4 Study design

A cross-sectional study design was employed, where data regarding parental satisfaction and PSOC were collected during a single clinical encounter. This design allowed for the assessment of parental perceptions immediately following the dental examination. The study had two phases. In the first phase, a sample of 130 participants was recruited between May 2019 and December 2021. This sample was used to conduct an exploratory factor analysis (EFA) to explore the factorial structure of the newly developed 13-item PSKTK questionnaire. Following this analysis, a second phase was conducted, recruiting a sample of 113 participants from April 2022 to February 2024. Confirmatory factor analysis (CFA) was performed to test the structural validity of the two factors estimated in the EFA phase.

### 2.5 Scale validation: EFA and CFA

EFA was conducted using Promax Oblique rotation with Kaiser normalization. Factor retention decision was based upon three methods (1): Eigenvalue (EV) > 1 (2); scree plot—random errors tend to converge on a linear line, and data points beyond the break point “the knee” are considered actual factors (19); and (3) parallel analysis—using a randomized dataset with an equal number of variables and an equal number of observations to obtain parallel EVs, only actual EVs that are larger than the parallel ones are considered actual factors (20). The EFA results revealed a two-factor structure: understanding and evaluation (7 statements) and confidence and comfort (6 statements). After the initial iteration, data reduction was performed according to the following guidelines: there was minimal loading in one item of 0.40, items were not loaded above.40 in more than one factor, there were no loading gaps smaller than.20 between factors, and items had commonalities >.50. A single item was dropped from the analysis because of a lack of minimal loading (.40) of the item in either factor. In the second iteration, the remaining 12 items subsequently converged into two factors with no further required data reductions (see Table 1 for details). The loading for the first factor (understanding and evaluation) was *EV* = *5.467*, Cronbach's *α* = .87, McDonald's Ω=0.87. The loading for the second factor (confidence and comfort) was *EV* = 1.56, Cronbach's *α* = .82, McDonald's Ω=0.82. Both factors together accounted for 58.55% of the common variance. The Pearson correlation coefficient between the two factors was significant and positive: *r* = .56, *p* < .001.

**Table 1:** Factor loadings of the “parental satisfaction with knee-to-knee” scale items based on an exploratory factor analysis.

Item number	Item	Loading in F1	Loading in F2
1	The dentist examined my children in the most professional manner	**0.889**	−0.088
2	The dentist put effort into reducing my child's anxiety about the examination	**0.831**	−0.042
3	The examination position allows the dentist to give me explanations during the examination	**0.781**	0.009
4	The dentist put effort into reducing my anxiety about the examination	**0.767**	−0.090
5	The examination position allows me to have a good view of my child's oral cavity	**0.687**	−0.030
6	In general, I am satisfied with the way my child was examined	**0.596**	0.202
7	The examination position allows me to better understand my child's dental condition	**0.547**	0.303
8	The examination position provides confidence to the child due to the physical contact with me	−0.085	**0.883**
9	The examination position provides confidence to the child due to the eye contact with me	−0.059	**0.882**
10	The examination position is more comfortable for the child compared to sitting in the dental chair	−0.105	**0.774**
11	In this examination position, the child is stable for the purpose of performing the examination	0.108	**0.635**
12	The examination position allows me to control the movements of the child's body and hands	0.200	**0.601**

Factor loadings > 0.50 are shown in bold to highlight salient loadings, indicating that the item substantially contributes to the respective factor.

**Figure 1:**
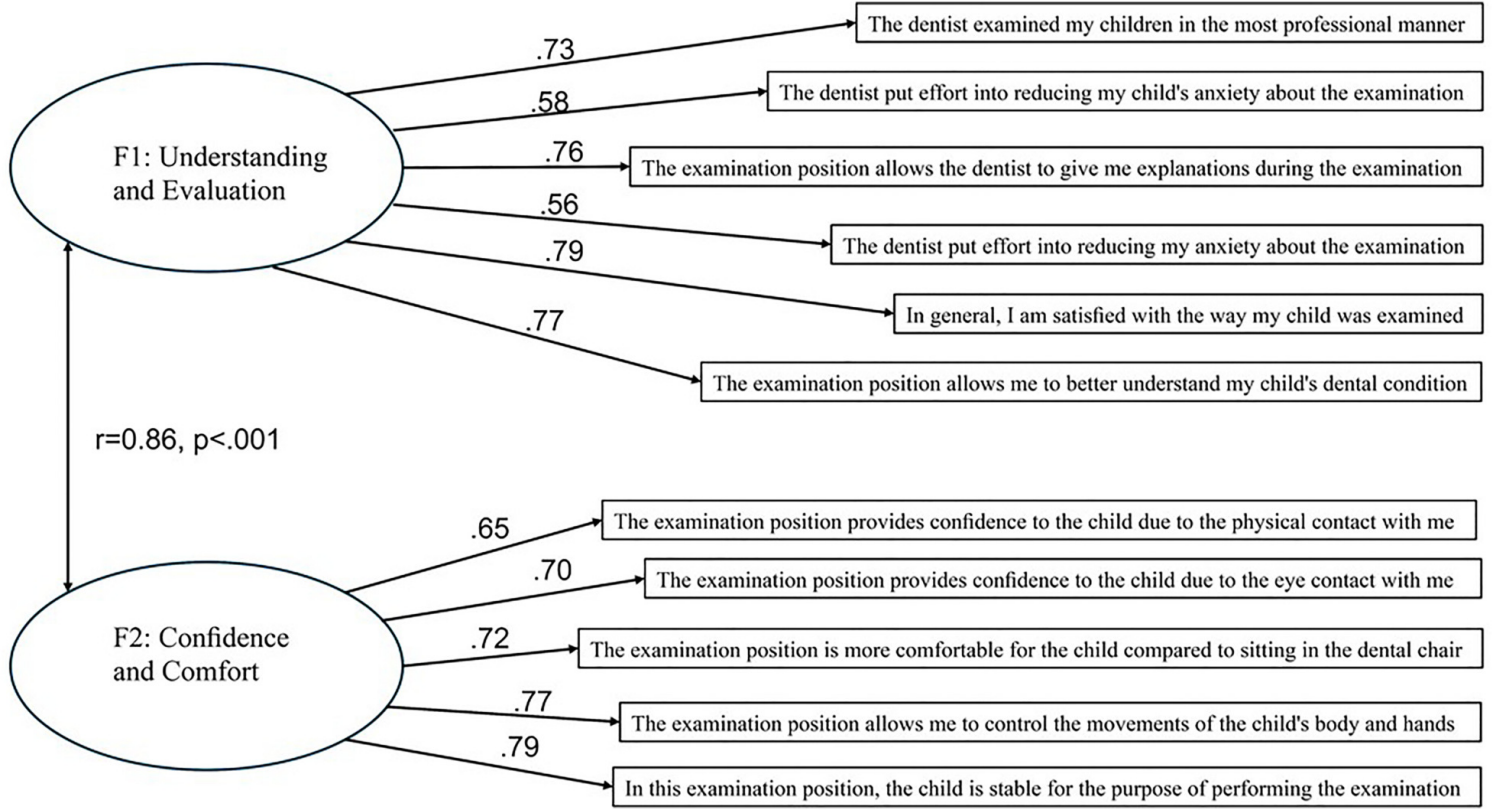
Standardized coefficients from the confirmatory factor analysis.

CFA using maximum likelihood estimation was performed twice on the new data. In the first model, we tested the two-factor structure estimated in the EFA phase. In the second model, we tested a competing one-factor solution, where all the items in the questionnaires loaded on one factor. The CFA was conducted via AMOS 29 (21). In accordance with Hoyle and Isherwood (22), the fit of the model to the data was evaluated via five goodness-of-fit indices. Two of these indices were absolute: the *χ*^2^ statistic and the root mean square error of approximation (RMSEA). The remaining three indices were incremental: the normed fit index (NFI), the comparative fit index (CFI) and the Tucker‒Lewis index (TLI). An RMSEA below.06 in combination with NFI, CFI, and TLI above.95 indicates excellent fit, whereas values below 0.08 and above.90 indicate adequate fit. Overall, the one-factor solution indicated a very poor fit of the model to the data [*χ*^2^(54) = 270.03, *p* < .001, *RMSEA* = .19, *CFI* = .73, *TLI* = .66, *NFI* = .68], whereas the two-factor solution indicated a much better fit to the data [*χ*^2^(37) = 85.80, *p* < .001, *RMSEA* = .11, *CFI* = .93, *TLI* = .90, *NFI* = .89]. Importantly, these values were obtained after omitting item 5 (“The examination position allows me to have a good view of my child's oral cavity”) from further analysis because of poor factor loading (.47). As such, all subsequent analyses were conducted without that specific item. Moreover, although the two-factor model demonstrated acceptable incremental fit (CFI = .93; TLI = .90), other indices indicated less optimal fit, including a significant *χ*^2^, RMSEA = .11, and NFI = .89. Importantly, the *χ*^2^ statistic is well known to be sensitive to sample size and minor model misspecifications, often leading to rejection even for reasonably fitting models (23). The elevated RMSEA and slightly reduced NFI may reflect characteristics common to psychological scale data. Specifically, the use of ordinal Likert-type items analysed with maximum likelihood estimation can inflate misfit indices when normality assumptions are violated (24). In addition, shared wording and conceptual overlap between items may introduce residual correlations not captured by the specified model. The factor loadings ranged from.56 to.82. The Cronbach's alpha for the first factor (understanding and evaluation) was 0.86, McDonald's Ω=0.85, and that for the second factor (confidence and comfort) was 0.87, McDonald's Ω=0.87. Figure 1 presents the standardized factor loadings for the model.

### 2.6 Statistical analysis

Descriptive statistics of the demographic variables are presented as absolute numbers and percentages for the categorical variables and as the means and standard deviations for the continuous variables. The scale factors were subsequently contrasted with other key variables of interest, such as PSOC, DKTK, age, and gender, and differences in the main variables were subsequently tested via correlations and mean differences tests: Pearson correlations, independent samples t tests, and Bonferroni correction for multiple comparisons. Descriptive statistics for the two-factor composites and PSOC (Table 2) revealed high means (M = 4.49–5.20, SD = .47–.65) with negative skewness (−.56 to −1.75) and positive kurtosis (.04–2.66), reflecting typical ceiling effects in positive-valence psychological scales. Despite moderate non-normality, parametric tests remain appropriate: averaged Likert scales approximate interval data, indicating central limit theorem applies (*N* = 113 > 30), and simulations confirm ANOVA/t-tests are robust to such skew/kurtosis (skew<|2|, kurtosis<|7|) even without transformation (25, 26). The data were analyzed via SPSS software (version 27.0.; SPSS, Inc., Chicago, Illinois, USA). The results were considered statistically significant at a *p* value < 0.05.

**Table 2:** Descriptive statistics.

Variable	M	SD	Min	Max	Skewness	Kurtosis
F1: Understanding and Evaluation.	4.69	.47	3.00	5.09	−1.75	2.66
F2: Confidence and Comfort	4.48	.65	1.80	5.05	−1.34	1.77
DKTK	4.60	.47	3.20	5.00	−.97	.01
PSOC	5.20	.56	3.42	6.05	−.56	.04

DKTK, Dentist's estimation of knee-to-knee; PSOC, parental sense of competence.

## 3 Results

### 3.1 Phase 1

The 130 examinations were performed by 15 dentists (12 females and 3 males). Most examinations (*n* = 106, 81.5%) were performed by female dentists. Regarding the participants, 90 of the accompanying parents (70.3%), and 64 of the children (49.2%) were females. Gender matching between the dentist and the parent occurred in 41 cases (32%). The age of the parents ranged from 22 to 55 years (M = 32.92, SD = 6.49), and the age of the children ranged from 1 to 4 years (M = 2.03, SD = 0.65). For 78 children (60%), this was their first dental visit.

The PSKTK and PSOC results are presented in Table 3. Approximately 90% of the parents were highly satisfied, and approximately 62% of the parents were classified as having high parental competence.

**Table 3:** Distribution of parental satisfaction with the knee-to-knee dental examination (PSKTK) score and parental sense of competence (PSOC) score.

Variable	Sample 1 (*n* = 130)		Sample 2 (*n* = 113)	
	Frequency	Percent	Frequency	Percent
PSKTK score				
1–2.99	1	0.8	1	0.9
3–3.99	12	9.2	16	14.1
4–5	117	90	96	85
PSOC score				
1–2.99	0	0	0	0
3–4.99	49	37.7	37	33.6
5–6	81	62.3	73	66.4

PSKTK, Parental satisfaction of knee-to-knee; PSOC, parental sense of competence.

To test the relationships of the PSKTK scale factors with the DKTK, PSOC, child's age and parent's age, a pairwise Pearson correlation was calculated. The full results are presented in Table 4. The results suggest that “Understanding and Evaluation” satisfaction was positively correlated with both the DKTK (*r* = .35, *p* < .001) and the PSOC (*r* = .26, *p* = .003). Similarly, “Confidence and Comfort” satisfaction also indicates a positive correlation with both the DKTK (*r* = .38, *p* < .001) and the PSOC (*r* = .29, *p* = .001). No other correlations were found to be significant.

**Table 4:** Descriptive statistics and correlation coefficients of the research variables (sample 1).

Variable	M	SD	1.	2.	3.	4.	5.
1. F1: Understanding and Evaluation.	4.68	.45					
2. F2: Confidence and Comfort	4.55	.56	.56***				
3. DKTK	4.58	.48	.35***	.38***			
4. PSOC	5.10	.58	.26**	.29**	.13		
5. Child's age	2.03	.65	.15	.06	.14	.10	
6. Parent's age	32.92	6.50	−.09	−.05	−.12	−.14	.09

DKTK. Dentist's estimation of knee-to-knee; PSOC.  parental sense of competence.

** *p* < .01.

*** *p* < .001.

To test for differences in the main research variables according to dentist sex, independent samples t tests were conducted. The full results are presented in Table 5. As shown in the table, the only significant difference found was for the DKTK (*t*(128) =  −3.53, *p* < .001, *Cohen's d* = .78). Specifically, female dentists (*M* = 4.65, *SD* = .46) had a greater estimation of parental satisfaction with the method than did male dentists (*M* = 4.28, *SD* = .49). No differences were found between children's genders for the research variables. No differences were found for the research variables based on gender concordance or discordance between the parent and the dentist. PSOC found to be significantly higher in cases of a child's first visit (M = 5.20, SD = .49) compared to children with previous dental experience (M = 4.94, SD = .67) (*t*(128) = −2.57, *p* = .01, *Cohen's d* = .44).

**Table 5:** Mean differences in the research variables: dentist's sex, child's sex, parent -dentist sex match and previous dental experience (Sample 1).

Groups	Male dentists (*n* = 3) Examinations = 24		Female dentists (*n* = 12) Examinations = 106			
Variable	*M*	*SD*	*M*	*SD*	*t*	*P Value*
F1: Understanding and Evaluation	4.67	.51	4.69	.44	−.22	.83
F2: Confidence and Comfort	4.44	.71	4.58	.51	−.90	.38
DKTK	4.28	.49	4.65	.46	−3.53	.001
PSOC	5.09	.50	5.10	.60	−.05	.96

	Boys (*n* = 66)		Girls (*n* = 64)			
Variable	*M*	*SD*	*M*	*SD*	*t*	*P Value*
F1: Understanding and Evaluation	4.68	.46	4.69	.45	−.02	.99
F2: Confidence and Comfort	4.56	.52	4.55	.59	.08	.94
DKTK	4.56	.50	4.60	.47	−.47	.64
PSOC	5.08	.64	5.12	.52	−.46	.64

	Gender concordance (*n* = 41)		Gender discordance (*n* = 87)			
Variable	*M*	*SD*	*M*	*SD*	*t*	*P Value*
F1: Understanding and Evaluation	4.68	.46	4.69	.45	−.02	.99
F2: Confidence and Comfort	4.56	.52	4.55	.59	.08	.94
DKTK	4.56	.50	4.60	.47	−.47	.64
PSOC	5.08	.64	5.12	.52	−.46	.64

	First visit (*n* = 78)		Previous dental experience (*n* = 52)			
Variable	*M*	*SD*	*M*	*SD*	*t*	*P Value*
F1: Understanding and Evaluation	4.69	.44	4.68	.47	−.18	.86
F2: Confidence and Comfort	4.59	.49	4.49	.65	−1.03	.30
DKTK	4.56	.48	4.61	.49	.61	.54
PSOC	5.20	.49	4.94	.67	−2.57	.01

DKTK, Dentist's estimation of knee-to-knee; PSOC, parental sense of competence.

### 3.2 Phase 2

A total of 113 examinations were performed by 21 dentists (15 females and 6 males). Most examinations were performed by female dentists (*n* = 73, 65.2%). Among the children, 47 (42.7%) were females while 66 (58.4%) of accompanying were females. In terms of the match between parents' and dentists' genders, discordance was observed 70 cases (63.1%). Parents' ages ranged from 22 to 49 years (M = 31.07, SD = 5.59), and children's ages ranged from 0.2 to 4.3 years (M = 2.17, SD = 0.76). For 49 children (43%), this was their first dental visit. In this sample, parents' sector was documented: 67 were ultraorthodox (62.6%), 28 were religious (26.2%), 6 were secular (5.6%), and 6 were “other” (5.6%). Out of the 113 participants, 66 provided the purpose of the visit. Among these, 34 (51.5%) presented due to dental trauma, 20 (30.3%) due to dental caries, 3 (4.5%) due to soft tissue pathology, and 9 (13.7%) for an initial routine dental examination.

According to Table 3, 85% of the parents reported high satisfaction. In terms of PSOC, 66.4% of the parents were classified as having high competence.

The Pearson correlations for the study variables with the continuous demographic variables, as well as the DKTK, are presented in Table 6. The analysis revealed several significant correlations between the variables. There was a strong positive correlation between “Confidence and Comfort” satisfaction and “Understanding and Evaluation” satisfaction (*r* = 0.72, *p* < 0.01). PSOC was also positively correlated with both “Understanding and Evaluation” satisfaction (*r* = 0.44, *p* < 0.01) and “Confidence and Comfort” satisfaction (*r* = 0.43, *p* < 0.01). Children's behavior during examination was positively associated with their DKTK (*r* = 0.21, *p* < 0.05) and age (*r* = 0.20, *p* < 0.05). Additionally, the child's anxiety was positively correlated with PSOC (*r* = 0.25, *p* < 0.05), the child's behavior (*r* = 0.48, *p* < 0.01), and “Understanding and Evaluation” satisfaction (*r* = 0.27, *p* < 0.01). Finally, there was a positive correlation between parent age and DKTK (*r* = 0.21, *p* < 0.05).

**Table 6:** Pearson correlation coefficients (sample 2).

Variable	M	SD	1	2	3	4	5	6	7
1. DKTK	4.60	.47							
2. F1: Understanding and Evaluation	4.69	.47	−.16						
3. F2: Confidence and Comfort	4.48	.65	−.14	.72**					
4. PSOC	5.20	.56	−.12	.44**	.43**				
5. Child's behavior	2.10	1.07	.21*	.09	.001	−.06			
6. Child's anxiety	2.86	1.48	.05	.27**	.17	.25*	.48**		
7. Age - Parent	31.07	5.58	.21*	.11	.02	−.05	.16	.05	
8. Age - Child	2.35	1.24	−.01	−.15	−.04	.02	.20*	−.06	.01

DKTK, Dentist's estimation of knee-to-knee; PSOC, parental sense of competence.

* *p* < .05.

** *p* < .01.

Furthermore, mean difference tests for the PSKTK scale factors and PSOC are presented in Tables 7–9. Initially, all the mean difference tests were conducted for the first factor, “Understanding and Evaluation” satisfaction (Table 7). The table reveals that male dentists had a greater estimation of satisfaction (M = 4.80, SD = 0.36) than female dentists did (M = 4.62, SD = 0.51). This difference was statistically significant, *t*(110) = 1.98, *p* = 0.05, with a medium effect size (Cohen's *d* = 0.39). Furthermore, significant differences were found among parents from different sectors [*F*(3, 103) = 3.44, *p* = 0.02, with an effect size of *η*^2^ = 0.09]. After applying the Bonferroni correction for multiple comparisons, the following results were obtained: secular were scored higher than “other” were (*p* = 0.02), whereas religious were scored lower than ultraorthodox were (*p* = .04). The ultraorthodox participants were also more highly scored than the “other” participants were (*p* = .025). No other comparison was found to be statistically significant. Similarly, all the mean difference tests were conducted for the second factor “Confidence and Comfort” satisfaction (Table 8). The table reveals that parents felt more confidence and comfort with male dentists (M = 4.66, SD = 0.51) than with female dentists (M = 4.37, SD = 0.70), *t*(110) = 2.32, *p* = 0.02, with a medium effect size (Cohen's *d* = 0.46). No other comparison was found to be statistically significant. Finally, significant differences were found among parents from different sectors in their PSOC scores [*F*(3, 103) = 3.441, *p* = 0.02, with an effect size of *η*^2^ = 0.09] (Table 9). After applying the Bonferroni correction for multiple comparisons, the following results were obtained: secular were scored higher than both religious (*p* = .01) and “other” (*p* = 0.01), and ultraorthodox were also more highly scored than “other” were (*p* = .04). All other mean difference tests conducted for PSOC were not significant.

**Table 7:** Mean comparison tests for F1: “understanding and evaluation” (sample 2).

Independent Variable	Subgroup	N	M	SD	t/F	p	Cohen's d/*η*^2^
Gender (Dentist)	Male	39	4.8	0.36	1.98	**0.05**	0.39
	Female	73	4.62	0.51			
Gender (Parent)	Male	47	4.68	0.49	−0.11	0.92	−0.02
	Female	66	4.69	0.46			
Gender (Child)	Male	63	4.68	0.47	0.03	0.97	0.01
	Female	47	4.68	0.48			
Gender Match	No	70	4.67	0.49	−0.32	0.75	−0.06
	Yes	41	4.7	0.46			
Previous dental experience	No	49	4.71	0.48	−0.67	0.5	−0.13
	Yes	61	4.65	0.47			
Sector (Parent)	Secular	6	4.93	0.15	3.44	**0.02**	0.09
	Religious	28	4.53	0.61			
	Ultraorthodox	67	4.75	0.36			
	Other	6	4.31	0.79			

Bold values indicate statistically significant results.

**Table 8:** Mean comparison tests for F2: “confidence and comfort” (sample 2).

Independent Variable	Subgroup	N	M	SD	t/F	p	Cohen's d/η^2^
Gender (Dentist)	Male	39	4.66	0.51	2.32	**0.02**	0.46
	Female	73	4.37	0.7			
Gender (Parent)	Male	47	4.51	0.63	0.44	0.66	0.08
	Female	66	4.45	0.67			
Gender (Child)	Male	63	4.43	0.68	−1.01	0.31	−0.19
	Female	47	4.56	0.62			
Gender Match	No	70	4.5	0.62	0.68	0.5	0.13
	Yes	41	4.41	0.71			
Previous dental experience	No	49	4.54	0.68	−1.04	0.3	−0.2
	Yes	61	4.41	0.64			
Sector (Parent)	Secular	6	4.93	0.16	1.76	0.16	0.2
	Religious	28	4.36	0.77			
	Ultraorthodox	67	4.5	0.6			
	Other	6	4.17	0.91			

Bold values indicate statistically significant results.

**Table 9:** Mean comparison tests for parents’ sense of competence (sample 2).

Independent Variable	Subgroup	N	M	SD	t/F	p	Cohen's d/η^2^
Gender (Dentist)	Male	39	5.12	0.54	1.47	0.14	0.29
	Female	73	4.94	0.63			
Gender (Parent)	Male	47	5.06	0.56	0.49	0.62	0.1
	Female	66	5.01	0.63			
Gender (Child)	Male	63	5.03	0.6	−0.1	0.92	−0.02
	Female	47	5.04	0.6			
Gender Match	No	70	5	0.6	−0.32	0.75	−0.06
	Yes	41	5.04	0.6			
Previous dental experience	No	49	5.09	0.55	−0.9	0.37	−0.18
	Yes	61	4.96	0.63			
Sector (Parent)	Secular	6	5.33	0.27	3.41	**.02**	.09
	Religious	28	4.94	0.66			
	Ultraorthodox	67	5	0.57			
	Other	6	4.67	0.61			

Bold values indicate statistically significant results.

## 4 Discussion

The 11-item PSKTK questionnaire developed for this study to assess parental satisfaction with the knee-to-knee dental examination position was found to be valid and reliable. This is the first study to assess parents' satisfaction with dental examinations in the knee-to-knee position and explore the correlation between parental sense of competence and dentists' estimation. The findings confirmed the study hypotheses.

In the present study, 90% of the parents in sample 1% and 85% of the parents in sample 2 expressed high satisfaction. In previous studies (8, 9), 82.5%–88% of dentists estimated that parents were satisfied with an examination via this method, and the current study confirms those estimations. A positive correlation was observed between the dentists' estimations (DKTK) and the parents' reported satisfaction (PSKTK). This suggests that dentists may have some ability to identify parents who will respond positively to this examination position. These parents might be more likely to actively participate, feel confident, and be comfortable during the examination.

This study revealed a correlation between PSOC and satisfaction with the knee-to-knee examination position. Parents with higher PSOC were more satisfied with a method requiring their active involvement, and vice versa. However, it is important to consider the role of clinical variables, such as the child's dental anxiety or behavioral cooperation during the visit. A child's distress can significantly influence parental satisfaction. In the current study, parents' perceptions of their child's anxiety, and child's behavior during the examination were both positively correlated with PSOC. These findings highlight the potential impact of parental competence and involvement on a child's dental experience and the establishment of a dental home. Parental roles in pediatric dentistry have undergone a significant transformation, evolving from passive involvement to active engagement, where engagement transcends task-specific participation to encompass a deeper emotional investment and shared responsibility in decision-making (27). This aligns with Marshman et al. (28), who reported a link between parental self-efficacy and their ability to monitor brushing habits and ensure twice-daily brushing.

The correlation between parents' satisfaction and PSOC is a good example of convergent validity (29). This is because both variables are logically linked to the concept of active parent participation. The lack of correlation between a child's age or parents' age and parents' satisfaction demonstrates discriminant validity (29). These variables (child's age and parents' age) are not directly connected to a parent's satisfaction with the examination position. These findings, together with those of the EFA and CFA, confirm that the PSKTK questionnaire is valid. It captures the intended concept and is not influenced by irrelevant factors such as the child's age or the parents' age.

The study revealed a correlation between dentists' sex and their assessment of parental satisfaction. Interestingly, this correlation differed between the two samples. In sample 1, female dentists rated parental satisfaction higher than males did, whereas the opposite was true in sample 2. It is important to consider dentists' sample sizes and other potential reasons behind the inconsistency between samples. Differences between male and female dentists were also reported by Crossley et al. (30), who reported gender variations in behavior guidance techniques performed by dentists, whereas Peretz et al. (31) did not find such differences.

The knee-to-knee examination requires physical proximity between the dentist and the parent, which may lead to discomfort in cases of gender discordance. Given that this study was conducted at a medical center serving a large religious and ultra-Orthodox population, the parental sector was documented in the second phase of the study. Despite the fact that the vast majority of participants (88.8%) identified as religious or ultra-Orthodox, parental satisfaction with the knee-to-knee position remained consistently high. Furthermore, no correlation was found between the gender of the dentist and the parent and the reported levels of satisfaction, even though gender matching was infrequent in most cases. These findings suggest that the perceived clinical necessity and professional nature of the examination may override cultural sensitivities regarding gender proximity in this specific pediatric context.

The study revealed significantly lower scores for PSOC and “Understanding and Evaluation” satisfaction scores for the “other” group than for the other sectors. Importantly, the “other” group was significantly smaller, and Hebrew might not have been their primary language, factors that likely influenced these results. This disparity highlights the persistent challenge of the language barrier and suggests that while physical proximity is tolerated, cognitive proximity, the ability to truly understand and evaluate the procedure, is the primary driver of parental competence and satisfaction. Parental perceptions of dental care are deeply embedded in cultural norms and healthcare expectations (32, 33); hence, the need for transcultural validation arises. A scoping review mapped the links between culture and behavior management strategies in pediatric dental settings. Cultural norms shaped communication styles. For instance, Latino families emphasized warm interpersonal interactions, whereas Pakistani families exhibited limited parental involvement due to language barriers (32). Simply translating existing instruments may overlook culturally specific nuances of parental engagement. Performing a rigorous transcultural adaptation ensures that the PSKTK scale maintains its conceptual integrity and psychometric robustness within a diverse cultural context.

This study has several limitations that warrant consideration. Since a convenience sample was used, the participants may not be fully representative of the broader population of parents seeking pediatric dental care. The sample was limited to those available at a specific clinical site, which may reflect a particular demographic or clinical sub-group. Non-representative sample limits the generalizability of the findings to other populations. This limitation specifically applies to the descriptive satisfaction results rather than the validation of the scale itself. The reliance on voluntary participation introduces a potential self-selection bias. It is possible that parents with stronger opinions, either highly satisfied or significantly dissatisfied, were more motivated to complete the questionnaire than the average parent. The cross-sectional nature of this study limits the ability to draw causal inferences. While we identified associations between parental satisfaction and their sense of competence, we cannot determine whether a positive dental experience improves parental competence or if confident parents are naturally more satisfied with the care provided. In addition, the examinations were performed by 23 different dentists, with each conducting a varying number of examinations (range: 1–29), which could introduce inter-operator variability. Finally, several variables that could influence parental satisfaction were not assessed in this study, including socioeconomic factors such as parental education, and child-specific factors like innate temperament, which can significantly modulate both child behavior and parental stress. These variables likely act as confounders that shape the PSOC and satisfaction. While the exclusion of these factors may limit the depth of our sociodemographic analysis, it does not undermine the fundamental validation of the PSKTK scale. Future studies examining parental satisfaction should incorporate these variables to further refine the understanding of these complex interactions.

In conclusion, the 11-item PSKTK questionnaire was found to be valid and reliable and can be used to assess satisfaction with knee-to-knee dental examination position among different populations. The majority of parents of toddlers were satisfied with this examination position, especially those with a greater sense of parental competence. Using the PSKTK scale to assess parental satisfaction can facilitate widespread adoption of the knee-to-knee position among diverse populations, ultimately contributing to improved oral health in toddlers.

## Data Availability

The raw data supporting the conclusions of this article will be made available by the authors, without undue reservation.
