# Supplementary material for: Development and validation of “parental satisfaction with knee-to-knee dental examination” scale: high correlation with parental sense of competence
Source: Front Oral Health. 2026 May 1;7:1822153. doi: 10.3389/froh.2026.1822153 (PMC13176141; doi:10.3389/froh.2026.1822153)
Supplement: Supplementary file 1 [file Datasheet1.pdf]

Item	Not agree at all	Not agree	Neutral	Agree	Absolutely agree
The examination position allows me to have a good view of my child's oral cavity	1	2	3	4	5
The examination position allows the dentist to give me explanations during the examination	1	2	3	4	5
The examination position allows me to better understand my child's dental condition	1	2	3	4	5
The examination position provides confidence to the child due to the physical contact with me	1	2	3	4	5
The examination position provides confidence to the child due to the eye contact with me	1	2	3	4	5
The examination position is more comfortable for the child compared to sitting in the dental chair	1	2	3	4	5
The examination position allows me to control the movements of the child's body and hands	1	2	3	4	5
In this examination position, the child is stable for the purpose of performing the examination	1	2	3	4	5
The dentist examined my children in the most professional manner	1	2	3	4	5
The examination position makes me uncomfortable due to the physical closeness with the dentist	1	2	3	4	5
In general, I am satisfied with the way my child was examined	1	2	3	4	5
The dentist put effort into reducing my anxiety about the examination	1	2	3	4	5

The dentist put effort into reducing my child's anxiety about the examination	1	2	3	4	5
